# Association of the neutrophil-to-lymphocyte ratio with sudden cardiac death in the patients with diabetic foot ulcer

**DOI:** 10.3389/fendo.2025.1697718

**Published:** 2025-11-12

**Authors:** Yi Chen, Junyan Zhao, Yuchen Sun, Zhongjing Yang, Caizhe Yang, Di Zhu

**Affiliations:** 1 Department of Endocrinology, Air Force Medical Center, Beijing, China; 2 Graduate School of China Medical University, Shenyang, China

**Keywords:** neutrophil-to-lymphocyte ratio, sudden cardiac death, diabetic foot ulcer, type 2 diabetes mellitus, diabetes mellitus

## Abstract

**Aims:**

This study aims to investigate the relationship between the neutrophil-to-lymphocyte (NLR) and the risk of sudden cardiac death (SCD) in the patients with diabetic foot ulcer (DFU).

**Methods:**

A retrospective study enrolled 688 patients with DFU who were admitted to Air Force Medical Center between January 2010 and December 2023. To control for potential confounding effects, a 1:1 propensity score matching (PSM) method was applied. The relationship between NLR and SCD risk was analyzed using the Kaplan-Meier (K-M) survival curve analysis, multivariate Cox proportional hazard regression model, Restricted cubic spline (RCS) model analysis and subgroup analyses.

**Results:**

Over a median follow-up period of 61 months, 38 cases of SCD were documented. Based on median NLR, participants were stratified into lower (<4.22) and higher (≥4.22) NLR groups. Cox proportional hazard model revealed that individuals with higher NLR was independently associated with the increased risk of SCD (HR: 3.64, 95% CI: 1.21 ~ 10.91, P=0.021). RCS model showed that SCD risk was non-linearly correlated with gradual increases in NLR levels. Subgroup analyses confirmed the stability of the results.

**Conclusions:**

Elevated NLR independently confers an increased risk for SCD in individuals with DFU.

## Introduction

Despite increasing awareness of risk factors and prevention strategies for type 2 diabetes mellitus (T2DM), the prevalence of T2DM continues to grow globally (1, 2). Diabetic foot ulcer (DFU) is one of the main complications of diabetes, with a lifetime risk of developing one estimated at 25% of all patients with diabetes (3). DFU refers to a wound caused by ischemia, infection, or impaired nerve conduction activity in the distal limb (4), which frequently leads to hospitalization for infection management or even amputation, imposing substantial physical, emotional, and economic burdens (5). Additionally, the 5-year mortality rate of DFU is around 30%, which posed a major threat to the life expectancy of patients with diabetes (6).

Sudden cardiac death (SCD) accounts for about 20% of total mortality in the general population, and is a prominent contributor to death among people with DFU (7, 8). SCD was defined as the unexpected natural death from a cardiac cause within a short time period, usually less than 1 hour from the onset of symptoms, in a person without any known prior condition that is fatal (9). Although numerous studies of patients with SCD have been conducted, research on SCD specifically in patients with DFU remains scarce (10, 11). Therefore, it is urgent to find predictive indicators for SCD in patients with DFU.

The neutrophil-to-lymphocyte ratio (NLR), easily derived from peripheral complete blood counts, is a novel hematological parameter for systemic inflammation and stress (12). As a reliable and available indicator of the immune response, it has been widely used in nearly every field of medicine, including sepsis, cancer, rheumatoid arthritis and metabolic syndrome (13–16). Previous research has revealed that the NLR has significant prognostic value in cardiovascular disease and even SCD (17–19).

However, the relationship between the NLR and the risk of SCD in patients with DFU remains less explored. Therefore, the objective of this study is to investigate the correlation between the NLR and the SCD risk, with the aim of providing helpful guidance for clinical practice in patients with DFU.

## Methods

### Study population

**Figure 1:**
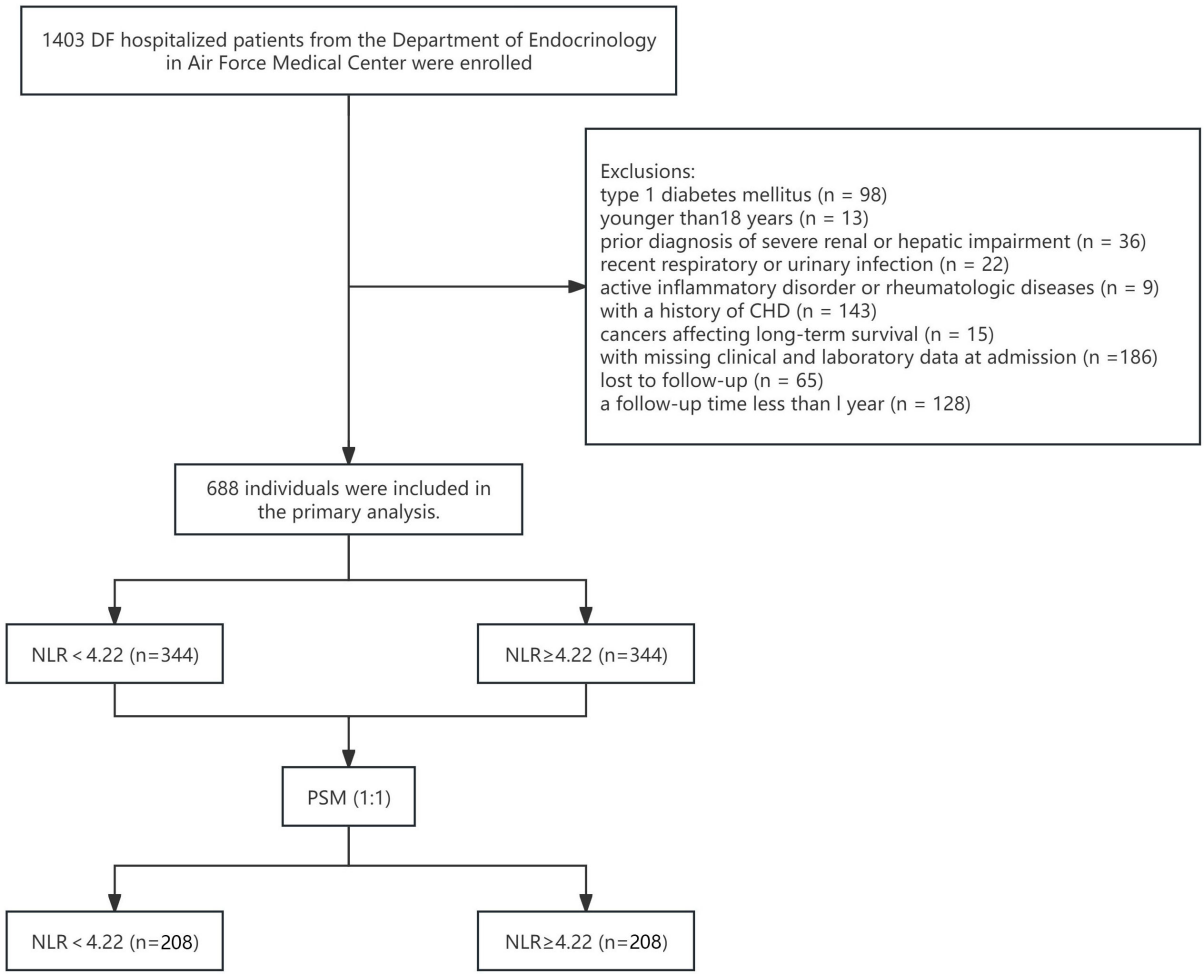
Flowchart for the patient selection.

In this retrospective study, 1,403 hospitalized patients with DFU were initially enrolled from the Department of Endocrinology in Air Force Medical Center, Beijing, China, between January 2010 and December 2023. The inclusion criteria included: (1) compliance with the diagnostic criteria for T2DM outlined by the American Diabetes Association (ADA), defined as a fasting plasma glucose (FPG)  ≥ 126 mg/dL (≥ 7.0 mmol/L), a two-hour oral glucose tolerance test value ≥ 200 mg/dL (≥ 11.1 mmol/L), or hemoglobin A1c (HbA1c) ≥6.5% (≥48 mmol/mol), and characterized predominantly by insulin resistance with relative insulin deficiency, or primarily by an insulin secretory defect with or without insulin resistance (20); (2) confirmation of DFU, defined as ulcerative lesions of the foot (including the ankle) associated with peripheral neuropathy, vascular disease, and infection (21). The exclusion criteria included: (1) type 1 diabetes mellitus (n = 98); (2) age younger than18 years (n = 13); (3) prior diagnosis of severe renal or hepatic impairment (n = 36); (4) acute infection that could significantly alter leukocyte counts, including recent respiratory or urinary tract infection (n = 22) and active inflammatory disorder or rheumatologic diseases (n = 9); (5) history of coronary heart disease (n = 143), to minimize confounding effects on SCD; (6) cancers affecting long-term survival (n = 15); (7) missing clinical and laboratory data at admission (n = 186), lost to follow-up (n = 65), a follow-up duration less than l year (n = 128). As a result, a total of 688 individuals were included in the primary analysis (**Figure 1**). To ensure the consent of participants, from September 1, 2024 to September 30, 2024, we conducted structured telephone interviews with the participants themselves whenever feasible. If a participant was unable to communicate, we contacted their family members or guardians instead. We provided detailed explanations of core information of this study, including key aspects such as research design, data collection methods, and privacy protection measures. After addressing their questions, we confirmed their voluntary consent, simultaneously verifying their survival status, documenting SCD occurrences, and recording any major adverse health events. This study was approved by the Air Force Medical Center Ethics Committee (Approval No. 2024-43-YJ01).

### Data collection

Clinical data were extracted from the electronic medical records of the hospital information system, including: (1) general demographic data [sex, age, body mass index (BMI), smoking status, drinking status]; (2) diabetes duration, DFU category; (3) comorbidities [history of cerebral infarction, hypertension and diabetic retinopathy (DR)]; (5) results of the first blood tests performed on admission [neutrophil (N), lymphocyte (L), HbA1c, FPG, total cholesterol (TC), triglycerides (TG), high-density lipoprotein cholesterol (HDL-C), low-density lipoprotein cholesterol (LDL-C), blood uric acid (BUA), serum creatinine (Scr) and blood urea nitrogen (BUN)]. The NLR was calculated as the ratio of neutrophil counts to lymphocyte counts.

### Endpoint

The main endpoint event was SCD, which was identified through hospitalization records (by ICD-10 code I46) and telephone follow-ups from September 1, 2024 to September 30, 2024. For each participant, the follow-up period began at the time of inclusion and ended upon the occurrence of SCD, death from other causes, or September 1, 2024—whichever came first.

### Propensity score matching

Given the differences in baseline characteristics among eligible patients with DFU across different NLR groups (**Table 1**), a cohort with comparable baseline characteristics was identified with Propensity Score Matching (PSM) method to minimize the effects of bias and confounding factors (12). In the PSM analysis, a logistic regression model was constructed to calculate the propensity score, with NLR served as an independent variable and all baseline parameters in **Table 1** included as covariates. These variables, encompassed age, sex, BMI, smoking status, drinking status, diabetes duration, DFU category, cerebral infarction, hypertension, DR, HbA1c, FPG, TC, TG, HDLC, LDLC, BUA, Scr and BUN, were incorporated into the propensity score calculation. The PSM analysis used a 1:1 nearest neighbor matching algorithm with a caliper width of 0.1. In order to assess the equilibrium of both groups, we calculated standardized mean difference (SMD) before and after matching. SMD of less than 0.10 indicated a well-balanced distribution across the matched groups.

**Table 1:** General clinical characteristics before and after PSM in two groups for NLR.

Variables	Before PSM				After PSM			
	Total (n=668)	Low-level(NLR<4.22) (n=334)	High-level(NLR≥4.22) (n=334)	P	Total (n=416)	Low-level(NLR<4.22) (n=208)	High-level(NLR≥4.22) (n=208)	P
Sex, n(%)				0.535				0.824
Male	497 (74.4)	245 (73.35)	252 (75.45)		306 (73.56)	152 (73.08)	154 (74.04)	
Female	171 (25.6)	89 (26.65)	82 (24.55)		110 (26.44)	56 (26.92)	54 (25.96)	
Age, year	63 (54, 71)	61(53, 69)	64(55, 72)	0.007	63 (55, 71)	63 (55, 71)	64(55, 71)	0.929
BMI, kg/m^2^	24.30 (22.50, 26.45)	24.23 (22.47, 26.65)	24.49 (22.60, 26.33)	0.481	24.49 (22.50, 26.60)	24.53 (22.48, 26.70)	24.39 (22.57, 26.45)	0.999
Smoking status, n(%)				0.949				0.915
Never	386 (57.78)	191 (57.19)	195 (58.38)		242 (58.17)	123 (59.13)	119 (57.21)	
Previous	30 (4.49)	15 (4.49)	15 (4.49)		19 (4.57)	9 (4.33)	10 (4.81)	
Current	252 (37.72)	128 (38.32)	124 (37.13)		155 (37.26)	76 (36.54)	79 (37.98)	
Drinking status, n(%)				0.895				1.000
Never	507 (75.9)	256 (76.65)	251 (75.15)		317 (76.2)	158 (75.96)	159 (76.44)	
Previous	16 (2.4)	8 (2.40)	8 (2.40)		7 (1.68)	4 (1.92)	3 (1.44)	
Current	145 (21.71)	70 (20.96)	75 (22.46)		92 (22.12)	46 (22.12)	46 (22.12)	
diabetes duration, year	15.00 (9.00, 20.00)	15.00 (8.00, 20.00)	16.00 (10.00, 22.00)	0.045	15.50 (10.00, 20.00)	15.00 (10.00, 20.00)	16.00 (10.00, 20.00)	0.954
DFU category				0.233				0.906
Non neuro-ischemic	36 (5.39)	12 (3.59)	24 (7.19)		13 (3.12)	7 (3.37)	6 (2.88)	
Neuropathic	233 (34.88)	120 (35.93)	113 (33.83)		139 (33.41)	67 (32.21)	72 (34.62)	
Ischemic	20 (2.99)	10 (2.99)	10 (2.99)		14 (3.37)	8 (3.85)	6 (2.88)	
Neuro-ischemic	379 (56.74)	192 (57.49)	187 (55.99)		250 (60.1)	126 (60.58)	124 (59.62)	
cerebral infarction, n (%)	155 (23.2)	79 (23.65)	76 (22.75)	0.783	98 (23.56)	50 (24.04)	48 (23.08)	0.817
hypertension, n (%)	424 (63.47)	201 (60.18)	223 (66.77)	0.077	269 (64.66)	135 (64.90)	134 (64.42)	0.918
DR, n (%)	352 (52.69)	183 (54.79)	169 (50.60)	0.278	204 (49.04)	103 (49.52)	101 (48.56)	0.844
HbA1c, %	9.10 (7.80, 11.10)	8.80 (7.50, 10.90)	9.50 (8.00, 11.20)	<0.001	9.10 (7.90, 11.10)	9.04 (7.90, 11.10)	9.30 (7.98, 10.95)	0.674
FPG,mmol/L	8.50 (6.58, 11.50)	7.70 (6.30, 9.90)	9.75 (7.00, 13.43)	<0.001	8.50 (6.68, 11.12)	8.30 (6.58, 10.83)	8.60 (6.70, 11.41)	0.468
TC,mmol/L	3.63 (2.98, 4.31)	3.74 (3.07, 4.40)	3.46 (2.90, 4.26)	0.008	3.57 (3.02, 4.31)	3.57 (2.98, 4.35)	3.59 (3.03, 4.29)	0.815
TG,mmol/L	1.25 (1.01, 1.68)	1.31 (1.03, 1.75)	1.23 (0.99, 1.60)	0.081	1.23 (1.00, 1.57)	1.24 (1.02, 1.58)	1.21 (0.99, 1.55)	0.387
HDL-C,mmol/L	0.83 (0.67, 1.00)	0.89 (0.74, 1.06)	0.77 (0.61, 0.93)	<0.001	0.81 (0.67, 0.97)	0.83 (0.69, 0.96)	0.81 (0.66, 0.98)	0.717
LDL-C, mmol/L	2.09 (1.63, 2.61)	2.12 (1.67, 2.63)	2.06 (1.63, 2.56)	0.200	2.12 (1.67, 2.64)	2.06 (1.66, 2.63)	2.17 (1.70, 2.65)	0.606
BUA,μmol/L	301.43 (233.00, 378.50)	306.00 (241.50, 374.92)	296.00 (229.00, 388.00)	0.584	289.50 (221.00, 364.50)	290.50 (220.50, 373.25)	286.50 (224.50, 357.25)	0.610
Scr,μmol/L	74.00 (57.00, 103.70)	70.00 (57.00, 89.88)	80.00 (58.25, 122.50)	<0.001	72.50 (57.00, 100.25)	71.00 (57.00, 97.00)	73.75 (56.75, 102.25)	0.623
BUN,mmol/L	6.30 (4.70, 9.10)	6.00 (4.60, 8.10)	6.70 (4.93, 10.30)	<0.001	5.90 (4.60, 8.50)	5.90 (4.60, 8.40)	6.00 (4.68, 8.80)	0.774
Neutrophil, ×10^9^/L	6.35 (4.33, 9.57)	4.40 (3.56, 5.71)	9.52 (7.30, 12.90)	<0.001	6.41 (4.50, 9.48)	4.60 (3.67, 6.10)	9.27 (6.97, 12.08)	<0.001
Lymphocyte, ×10^9^/L	1.45(1.12,1.94)	1.81(1.40,2.30)	1.20(0.94,1.50)	<0.001	1.43 (1.15, 1.90)	1.75 (1.39, 2.19)	1.28 (0.99, 1.51)	<0.001
Clinical outcomes								
SCD, n (%)	38 (5.69)	10 (2.99)	28 (8.38)	0.003	17 (4.09)	6 (2.88)	11 (5.29)	0.216

PSM, propensity score matching; NLR, neutrophil-to-lymphocyte ratio; BMI, body mass index; DR, diabetic retinopathy; HbA1c, hemoglobin A1c; FPG: fasting Plasma glucose; TC, total cholesterol; TG, triglycerides; HDL-C, high density lipoprotein cholesterol; LDL-C, low-density lipoprotein cholesterol; Scr, serum creatinine; BUA, blood uric acid; BUN, blood urea nitrogen; SCD, sudden cardiac death.

### Statistical analysis

All statistics were analyzed with R statistical software (R version 4.2.2), SPSS Statistics 26 or PASS 15.0 software. The power analyses were conducted using the “Tests for Two Survival Curves using Cox’s Proportional Hazards Model” in PASS 15.0 software, with a two-sided significance level (α) of 0.05 and a target power (1-β) of 0.8. According to our preliminary data, the cumulative incidence of SCD over a follow-up period of 5 years was 0.03 in the lower NLR group and 0.09 in the higher NLR group, corresponding to a hazard ratio (HR) of 3.0. Assuming a 1:1 sample allocation ratio between the two groups and accounting for a 10% loss to follow-up, the calculation indicated that a minimum total sample size of 454 participants (approximately 227 per group) is required to achieve a statistical power of 0.80069. This study enrolled a total of 688 patients, which exceeds the calculated minimum sample size.

Continuous variables were depicted as mean ± standard deviation (SD) for normally distributed data or median [interquartile range (IQR): 25th-75th percentile] for non-normally distributed data. Differences between groups were analyzed using the t-test for normally distributed data or Mann-Whitney U-test for non-normally distributed data. Categorical variables were documented as counts with their respective proportions and compared using the Chi-square test or Fisher’s exact test. All patients were separately segregated into two groups according to median NLR. The Kaplan-Meier (K-M) curves were employed to visualize the stability of HR in survival analysis and evaluate the SCD risk for individuals with DFU at different NLR levels, with the log-rank test applied for comparisons. Univariate and multivariate analyses were performed using the Cox proportional hazards model to investigate the unique correlations between NLR and risk of SCD by Schoenfeld residuals test and the Grambsch-Therneau test. The models were stratified into four levels to control for possible confounding factors. The results were reported as HR with 95% confidence intervals (CI). Restricted cubic splines (RCS) were applied to explore potential non-linear relationships between NLR and SCD risk and identify inflection points; the model was selected based on the lowest Akaike Information Criterion (AIC) value and included four knots. A subgroup analysis was employed to examine the effect of NLR on SCD risk within different subgroups. Stratification was carried out using a Cox regression model according to the following variables: sex, age (<65 and ≥65 years), BMI (<24 and ≥24 kg/m^2^), smoking status, drinking status, diabetes duration (<15 and ≥15 years), DFU category (Non neuro-ischemic, Neuropathic, Ischemic and Neuro-ischemic), cerebral infarction, hypertension and DR. All statistical analyses were two-sided, and a p-value < 0.05 was considered statistically significant.

## Results

### Baseline characteristics of subjects

In the unmatched cohort, a total of 688 participants with DFU were finally included in this retrospective analysis. The participants were with a median age of 63 years old, with males accounting for 74.4% and females 25.6% of the study population. Meanwhile, no cases of foot deformity, including Charcot foot were documented in this patient cohort. All patients were assigned into two groups according to the median NLR, including a lower NLR group (NLR < 4.22) and a higher NLR group (NLR ≥ 4.22).

Before PSM, compared with the lower NLR group, patients with higher NLR were characterized by an older median age (61 vs.64 years old, P = 0.007) with longer diabetes duration (15 vs.16 years old, P = 0.045). Additionally, regarding clinical parameters, the higher NLR group exhibited higher HbA1c [8.80 (7.50, 10.90) vs.9.50 (8.00, 11.20)%, p < 0.001], FPG [7.70 (6.30, 9.90) vs.9.75 (7.00, 13.43) mmol/L, p < 0.001], Scr [70.00 (57.00, 89.88) vs.80.00 (58.25, 122.50) μmol/L, p < 0.001], BUN [6.00 (4.60, 8.10) vs.6.70 (4.93, 10.30) mmol/L, p < 0.001] and lower TC [3.74 (3.07, 4.40) vs.3.46 (2.90, 4.26) mmol/L, p < 0.001], HDL-C [0.89 (0.74, 1.06) vs.0.77 (0.61, 0.93) mmol/L, p < 0.001]. As for clinical outcomes, the high NLR group showed higher SCD incidence [28 (8.38%) vs.10 (2.99%), p =0.003]. A 1: 1 PSM analysis was performed in order to normalize the differences in baseline characteristics, resulting in 208 well-matched pairs. Demographics, comorbidities, and laboratory parameters showed equilibrium among the post-PSM cohorts. After PSM, the higher NLR group didn’t show higher SCD incidence [11 (5.29) vs.6 (2.88), p =0.216]. More detailed results can be found in **Table 1**.

### Association of the higher NLR with higher risk of SCD

**Figure 2:**
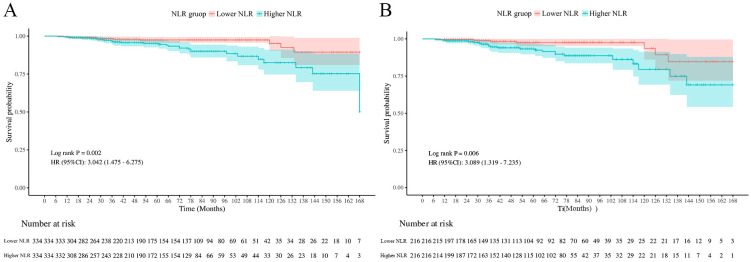
Kaplan–Meier curves for SCD risk before **(A)** and after **(B)** propensity score matching.

Throughout the median duration of follow up of 61 months (range: 1–14 years), 81 (11.8%) fatalities were recorded among 688 individuals, of which 38 (5.5%) were due to SCD. K-M analysis demonstrated a significantly positive association between higher NLR and increased SCD risk (P value=0.002; **Figure 2A**). Additionally, K-M survival curves comparing the two groups (NLR < 4.22 vs. NLR ≥ 4.22) highlighted that even after PSM, patients with a NLR ≥ 4.22 consistently demonstrated significantly higher SCD risk compared to patients with a lower NLR (P value=0.006; **Figure 2B**).

In order to clarify the potential relationship between the NLR and SCD incidence in patients suffering from DFU, both univariate and multivariate Cox regression models were performed, with NLR classified as binary. As illustrated in **Table 2**, in the crude Cox regression model without adjustments (model 1), a heightened NLR (≥ 4.22) was demonstrably linked with SCD incidence (HR: 3.04, 95% CI: 1.47 ~ 6.27, P = 0.003). However, no significant relations were discovered between NLR and SCD in patients with DFU in model 2,3 and 4 while adjusting for confounding variables. After PSM and multivariate adjustment, the risk of SCD significantly increased with higher NLR value (model4, HR: 3.64, 95% CI: 1.21 ~ 10.91, P = 0.021). Each one-unit increase in NLR was associated with a 85% increased risk of SCD (model4, HR: 1.85, 95% CI: 1.05 ~ 3.26, P = 0.033). Detailed data are presented in **Table 3**.

**Table 2:** Univariate and multivariate Cox proportional hazard models of NLR with risk of SCD before PSM.

NLR Group (n=668)	Model 1		Model 2		Model 3		Model 4	
	HR (95%CI)	*P*	HR (95%CI)	*P*	HR (95%CI)	*P*	HR (95%CI)	*P*
Lower NLR (<4.22) (n=334)	1.00 (Reference)		1.00 (Reference)		1.00 (Reference)		1.00 (Reference)	
Higher NLR (≥4.22) (n=334)	3.04 (1.47 ~ 6.27)	0.003	1.88 (0.88 ~ 4.03)	0.103	1.62 (0.74 ~ 3.56)	0.228	1.21 (0.58 ~ 2.55)	0.608
Per SD increase	1.31 (1.19 ~ 1.43)	<0.001	1.27 (1.12 ~ 1.43)	<0.001	1.19 (1.03 ~ 1.37)	0.015	1.20 (0.99 ~ 1.46)	0.069

Model1: Crude.

Model2: Adjust: sex, age, BMI, smoking status, drinking status.

Model3: Adjust: sex, age, BMI, smoking status, drinking status, diabetes duration, DFU category, cerebral infarction, hypertension, DR.

Model4: Adjust: sex, age, BMI, smoking status, drinking status, diabetes duration, DFU category, cerebral infarction, hypertension, DR, HbA1c, FPG, TC, TG, HDLC, LDLC, BUA, Scr and BUN.

NLR, neutrophil-to-lymphocyte ratio; SCD, sudden cardiac death; PSM, propensity score matching; HR, Hazard Ratio, CI, Confidence Interval; BMI, body mass index; DR, diabetic retinopathy; HbA1c, hemoglobin A1c; FPG: fasting Plasma glucose; TC, total cholesterol; TG, triglycerides; HDL-C, high density lipoprotein cholesterol; LDL-C, low-density lipoprotein cholesterol; Scr, serum creatinine; BUA, blood uric acid; BUN, blood urea nitrogen.

**Table 3:** Univariate and multivariate Cox proportional hazard models of NLR with risk of SCD after PSM.

NLR Group (n=416)	Model 1		Model 2		Model 3		Model 4	
	HR (95%CI)	*P*	HR (95%CI)	*P*	HR (95%CI)	*P*	HR (95%CI)	*P*
Lower NLR (<4.22) (n=208)	1.00 (Reference)		1.00 (Reference)		1.00 (Reference)		1.00 (Reference)	
Higher NLR (≥4.22) (n=208)	1.75 (0.65 ~ 4.75)	0.270	1.07 (0.36 ~ 3.18)	0.897	1.66 (0.59 ~ 4.69)	0.339	3.64 (1.21 ~ 10.91)	0.021
Per SD increase	1.15 (0.94 ~ 1.41)	0.173	1.63 (0.99 ~ 2.68)	0.054	1.37 (0.68 ~ 2.76)	0.375	2.03 (1.13 ~ 3.64)	0.018

Model1: Crude.

Model2: Adjust: sex, age, BMI, smoking status, drinking status.

Model3: Adjust: sex, age, BMI, smoking status, drinking status, diabetes duration, DFU category, cerebral infarction, hypertension, DR.

Model4: Adjust: sex, age, BMI, smoking status, drinking status, diabetes duration, DFU category, cerebral infarction, hypertension, DR, HbA1c, FPG, TC, TG, HDLC, LDLC, BUA, Scr and BUN.

NLR, neutrophil-to-lymphocyte ratio; SCD, sudden cardiac death; PSM, propensity score matching; HR, Hazard Ratio, CI, Confidence Interval; BMI, body mass index; DR, diabetic retinopathy; HbA1c, hemoglobin A1c; FPG: fasting Plasma glucose; TC, total cholesterol; TG, triglycerides; HDL-C, high density lipoprotein cholesterol; LDL-C, low-density lipoprotein cholesterol; Scr, serum creatinine; BUA, blood uric acid; BUN, blood urea nitrogen.

### A non-linear correlation between NLR and risk of SCD

**Figure 3:**
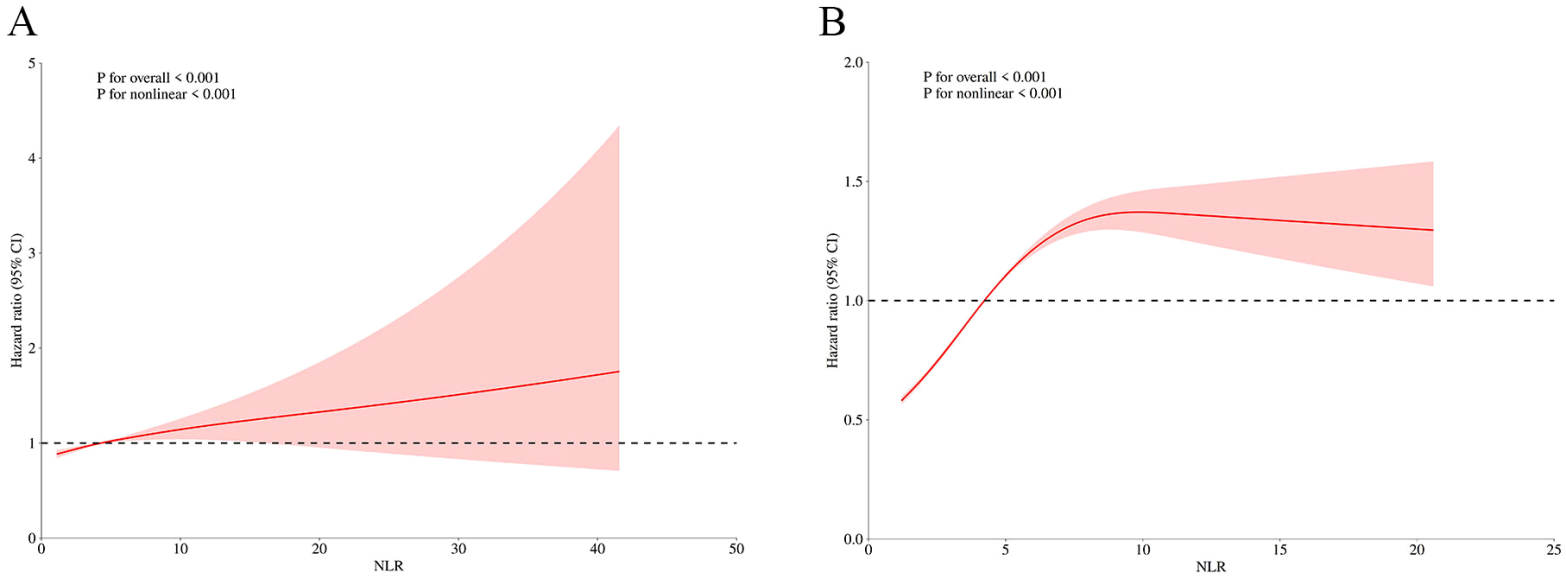
Underlying non-linear correlations with SCD risk before **(A)** and after **(B)** propensity score matching. The association was adjusted for sex, age, BMI, smoking status, drinking status, diabetes duration, DFU category, cerebral infarction, hypertension, DR, HbA1c, FPG, TC, TG, HDLC, LDLC, BUA, Scr and BUN.

In addition, we also analyzed the original data when the NLR was treated as continuous variables, using RCS analysis to explore potential non-linear relationships between NLR and SCD risk. Based on smooth curve fitting and a generalized additive model, the threshold of the NLR on SCD risk was studied and the inflection point was identified. After adjusting for interfering factors, non-linear correlation was found between NLR and SCD risk with an inflection point at 4.22 before and after PSM (P for nonlinear<0.001,P for overall<0.001 for both, **Figure 3**). Notably, beyond this inflection point (NLR ≥ 4.22), the risk of SCD elevated significantly as the NLR increased.

### Subgroup analysis

**Figure 4:**
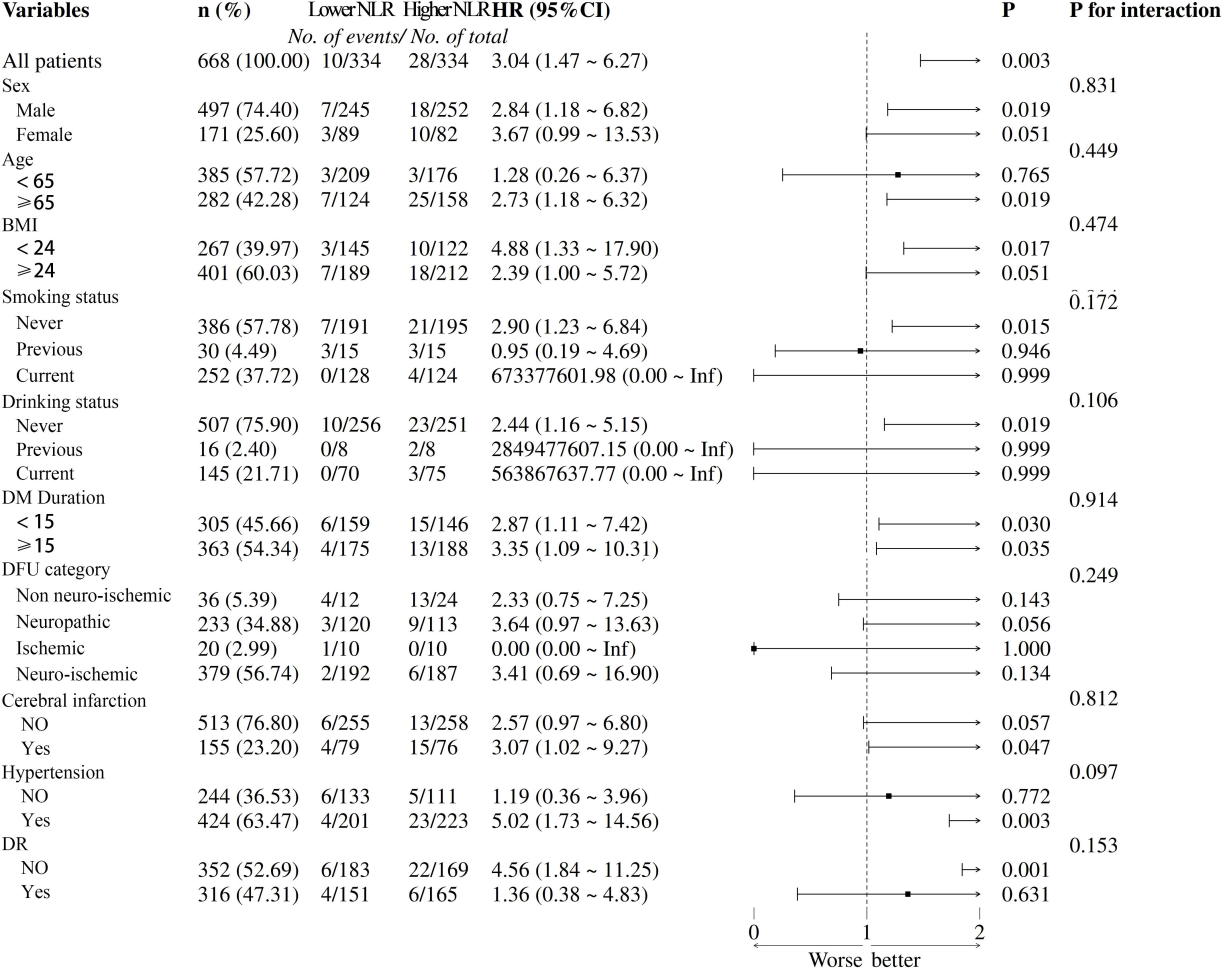
Subgroup analysis for the effect of the NLR on SCD risk.

A subgroup analysis was carried out to investigate the relationship between the NLR and SCD risk in patients with DFU based on sex, age (<65 and ≥65 years), BMI (<24 and ≥24 kg/m^2^), smoking status, drinking status, diabetes duration (<15 and ≥15 years), DFU category (Non neuro-ischemic, Neuropathic, Ischemic and Neuro-ischemic), cerebral infarction, hypertension and DR. The results showed that there was a consistent association between the increasing NLR and the higher risk of SCD in all subgroups (**Figure 4**). There were no significant stratification factors affecting the relationship between the NLR and SCD risk.

## Discussion

To our knowledge, this is the first study to comprehensively explore the relationship between the NLR and risk of SCD using multiple methods among patients with DFU. Through the analysis of varieties of data from 688 participants with DFU, we revealed that the elevated NLR levels are significantly correlated with an increased risk of SCD. These findings maintained consistent across subgroup analyses. Collectively, the results of this study provide convincing evidence that NLR could serve as a sensitive and valuable predictor for SCD in routine clinical practice in patients with DF.

NLR, as an indicator that integrates two immune pathways — natural immunity (via neutrophils) and acquired immunity (via lymphocytes), has proven to be more predictive than single parameter of neutrophil or lymphocyte (22). Numerous studies have confirmed that inflammatory and immune mechanisms play crucial roles in the pathogenesis and progression of DFU, especially with regard to its long-term prognosis (23, 24). Furthermore, NLR has demonstrated a predictive value for the mortality of cardiovascular diseases, including hypertension, heart failure and coronary heart disease (25–27). Potential explanations of the NLR as a marker for predicting SCD are as follows: (1) Elevated NLR may exacerbate inflammatory activity and act as a critical factor in atherosclerosis progression, including increasing plaque instability and plaque rupture (28); (2) A large number of inflammatory mediators secreted by neutrophils could modulate ion channel function and produce arrhythmias (29); (3) Inflammation could enhance sympathetic tone, which is associated with reduced heart rate variability, particularly in patients with diabetes, thus resulting in tachycardia and electrical instability of the heart (30). Taken together, these multiple physiological mechanisms contribute to the cardiovascular dysfunction, culminating in the increased risk of SCD.

As a serious complication of diabetes, DFU is characterized by unique clinical features and complex pathophysiological mechanisms (31). The development of DFU depends on the complex interaction of hyperglycemia, inflammation, and oxidative stress. Overproduction of reactive oxygen species induced by hyperglycemia significantly contributes to endothelial dysfunction and inflammation (32). A growing number of evidence has confirmed that DFU is characterized by high incidence, amputation rate, recurrence rate and mortality rate, making it a critical global healthcare challenge (33, 34). SCD remains one of the most perilous and unpredictable complications for patients suffering from DFU (35). Mechanistically, acute hypoglycemia or electrolyte disturbances, especially hyperglycemia, can mediate fatal arrhythmias through cardiac autonomic activity (36, 37). At the same time, atherosclerosis, endothelial dysfunction, platelet aggregation, thrombosis, inflammation and immune mechanism disorders are easy to cause myocardial ischemia (38). Together, these elements exacerbate the appearance of SCD. Due to cardiac autonomic neuropathy, patients with DFU often suffer from damage to the cardiac sensory afferent nerves, which significantly increases their pain tolerance (39). As a result, when these patients experience a cardiac event, such as a myocardial infarction, they usually do not exhibit the typical chest pain. Instead, they may present only with atypical symptoms, such as a mild chest discomfort, fatigue, dizziness, or nausea, which is known as a silent myocardial infarction (40, 41). This can easily prevent patients from identifying potential health crises and result in missed or delayed diagnoses, consequently missing timely treatment and increasing the risk of SCD (42). Despite advancements in medical care and treatment strategies for DFU, the risk of SCD looms large due to its abrupt onset and the difficulty in accurate risk prediction, highlighting the urgent need for comprehensive predictors (43).

Therefore, the NLR plays a vital role in predicting the clinical prognosis, especially SCD, in individuals with DFU. Despite the close association between NLR, SCD and DFU, no previous studies have focused on the role of NLR in evaluating and predicting the incidence of SCD in patients with DFU. A prospective observational analysis conducted by Ozyilmaz et al. demonstrated that patients with a predicted five-year SCD risk above 6% had notably higher NLR levels. Specifically, their NLR averaged 2.4 ± 1.8, compared to 1.8 ± 0.6 in those with a five-year SCD risk of ≤5.9% in hemodialyzed patients (19). Previous researches exploring predictors of SCD typically focused on the population with CVD, while relatively few studies target this specific population of patients with DFU. Given that patients with DFU are themselves at high cardiovascular risk, it has important clinical implications to explore the predictive value of the NLR with SCD in this particular population. In the present study, the NLR value indicates a significant difference in SCD between the higher and lower NLR groups when the NLR cutoff is set to the median 4.22 (**Figure 2** and **Tables 2**, **3**). These results suggest that neutrophils and lymphocytes, as key components, play an important role in chronic inflammation and immune responses throughout the entire process of DFU. Thus, monitoring NLR levels in clinical practice may help in early identification and intervention for risks associated with SCD, thereby improving prognosis in patients with DFU.

The association between NLR and SCD risk aligns with broader research on NLR as a cardiovascular prognostic marker, though NLR cutoff values vary across studies based on population characteristics and clinical endpoints. A study including 3, 251 participants with diabetes identified an NLR cutoff of 3.48 as predictive of all-cause and cardiovascular mortality in patients with diabetes, except for cardiovascular mortality in patients under 60 years old (44). Similarly, a prospective cohort study found that individuals with NLR levels above 2.48 had a significantly higher risk of mortality from any cause (37%) and cardiovascular disease (63%) compared to those with lower NLR levels in patients with diabetes (45). In the present study, the NLR cutoff of SCD in patients with DFU identified in this study was 4.22, which was significantly higher than that in other studies on diabetes-related cardiovascular risk. In addition to chronic diabetic inflammation, patients with DFU also experience persistent irritation from foot ulcers, leading to more pronounced neutrophilia and lymphopenia with inflammatory imbalance (46). Furthermore, the synergistic amplification of cardiovascular risks by DFU and vascular lesions exacerbates immune imbalance (47). Therefore, a higher NLR cutoff is required to define the risk of SCD.

The main strength of this research is that the NLR can be applied to different clinical phases, grades and even basic level hospitals in less developed areas, due to its convenience and affordability. Moreover, the diversity of analytical methods and the longer follow-up period make the results more robust. However, this present research still needs to be improved. First, because of the dynamic and long-term progression of DFU and its complications, including only baseline data in the analysis can lead to a bias in the results. Second, we do not necessarily have an exhaustive range of adjusted confounding factors, allowing for possible confounders that may have an impact on the association of NLR with SCD, such as renal function markers including albumin/creatinine ratio (ACR), well-established risk markers for SCD, including left ventricular ejection fraction, NT-proBNP, and troponin levels and medications, such as statins, renin-angiotensin-aldosterone system (RAAS) inhibitors, and antidiabetic therapies. Thirdly, the inherent defect of a single center retrospective study makes it possible to select and sample bias. Finally, we adopted the cohort-specific median to divide participants into two groups, which may restrict the generalization of our findings to other cohorts or real-world clinical settings. Therefore, future research should reference more diverse and evidence-based grouping bases. Meanwhile, the findings of this study need to be confirmed by a randomized, double-blind, multi-center, prospective longitudinal cohort study.

## Data Availability

The raw data supporting the conclusions of this article will be made available by the authors, without undue reservation.
