# Correction: Association of the neutrophil-to-lymphocyte ratio with sudden cardiac death in the patients with diabetic foot ulcer

**DOI:** 10.3389/fendo.2026.1780296

**Published:** 2026-01-15

**Authors:** Yi Chen, Junyan Zhao, Yuchen Sun, Zhongjing Yang, Caizhe Yang, Di Zhu

**Affiliations:** 1 Department of Endocrinology, Air Force Medical Center, Beijing, China; 2 Graduate School of China Medical University, Shenyang, China

**Keywords:** neutrophil-to-lymphocyte ratio, sudden cardiac death, diabetic foot ulcer, type 2 diabetes mellitus, diabetes mellitus

In the **Abstract**, there was an incorrect sentence: “Based on median NLR, participants were stratified into higher (<4.22) and lower (≥4.22) NLR groups”. This has been corrected to read: “Based on median NLR, participants were stratified into lower (<4.22) and higher (≥4.22) NLR groups.”

The original version of this article has been updated.

